# Supplementary material for: Liupao Tea Extract Alleviates Rheumatoid Arthritis in Mice by Regulating the Gut–Joint Axis Mediated via Fatty Acid Metabolism
Source: Foods. 2025 Aug 18;14(16):2854. doi: 10.3390/foods14162854 (PMC12385620; doi:10.3390/foods14162854)
Supplement: Supplementary file 1 [file foods-14-02854-s001.zip › foods-3762687-supplementary.pdf]

Liupao Tea Extract Alleviates Rheumatoid Arthritis in Mice by Regulating the Gut–Joint Axis Mediated via Fatty Acid Metabolism

Supplementary methods

1. Gut microbiota analysis

The V3 - V4 regions of the bacterial 16S rRNA genes were amplified using universal primers 343F (5'-TACGGRAGGCAGCAG-3') and 798R (5'-AGGGTATCTAATCCT-3'). The PCR-amplified library underwent purification with Agencourt AMPure XP beads and was subjected to a second round of PCR amplification. After purification, the final amplicon was quantified using the Qubit dsDNA assay kit (Life Technologies, Q32854). The purified amplicon was pooled in equal proportions for sequencing. Subsequently, 16S rRNA gene sequencing of the fecal DNA was conducted on an Illumina MiSeq platform (MiSeq PE300, Illumina, USA).

Following filtration and chimera removal, clean sequences were ultimately acquired. The QIIME2 package was utilized to select the representative read for each Amplicon Sequence Variant (ASV). Subsequently, α-diversity and β-diversity indices were computed using the QIIME2 software. The assessment of microbial community structure was then conducted based on the outcomes of these analyses.

2. Preparation and analysis of short-chain fatty acids (SCFAs)

SCFA extraction: 90 mg of fecal samples were mixed with 300 μl OmniSolv pure water and vortex for 20 s, repeated three times using vortex mixer. The mixture was then incubated at 4°C with shaking for 30 mins, followed by centrifugation at 13,000 × g for 30 mins. 200 μl of supernatant (fecal homogenate) was transferred into a new microtube pre-added with 10 μl of 5 M HCl to adjust the pH to 2. The acidified supernatant samples were extracted by adding 100 μl of anhydrous diethyl ether (DE) (2:1, *v/v*), vortexed and incubated on ice for 5 mins, and then centrifuged for 5 mins at 10,000 × g. The DE layer (containing SCFAs) was transferred to a new microtube containing anhydrous Na_2SO_4 to remove the residual water. The remaining aqueous layer was further extracted with DE for two more times. The DE layers were pooled

and mixed for further derivatization.

Derivatization procedure: 100 µl of DE extract was accurately transferred into a glass insert in a GC vial and 5 µl of BSTFA was added. The mixture was kept in the GC vial and incubated at 37°C for 2 hours. Then, derivatized samples were loaded onto GC-MS. Pure water was used as a blank sample to correct the background. The blank sample was processed using the same procedure as the fecal samples.

GC-MS analysis was conducted using a 7250-gas chromatograph/8890 mass selective detector (Agilent Technologies, Santa Clara, CA, USA) equipped with an HP-5 ms capillary column (30 m × 0.25 mm × 0.25 µm film thickness) (Agilent Technologies). The injector, ion source, quadrupole, and the GC-MS interface temperature were 260, 230, 150, and 280°C, respectively. The flow rate of the helium carrier gas was maintained at 1 mL/min. A volume of 1 µl of the derivatized sample was injected with a solvent delay time of 3 mins and a split ratio of 10:1. The initial column temperature was set at 40°C, kept constant for 2 mins, then increased to 150°C at a rate of 15°C/min, maintained for 1 min, and finally raised to 280°C at a rate of 30°C/min, where it was held for 5 mins. The ionization of analytes was conducted in the electron impact (EI) mode at 70 eV.

Table S1. Chromatographic conditions for LPTE analysis.

Time (min)	Flow rate (mL/min)	Mobile phase A (%)	Mobile phase B (%)	Injection volume (μL)
0	0.4	98	2	5
2	0.4	98	2	
13	0.4	0	100	
18	0.4	0	100	
20	0.4	98	2	

Analytical conditions: Waters Acquity UPLC HSS T3 (1.8 μm, 2.1 mm × 100 mm) 40°C column temperature. Mobile phases: A was ultrapure water and B was acetonitrile, both of which contained 0.1% formic acid in ESI+ and ESI- modes.

Table S2. Mass spectrometric conditions for LPTE and serum metabolomic analysis.

Parameters	Values
Capillary voltage	2000 V
Cone	40 V
Source temperature	150°C
Desolvation temperature	500°C
Cone gas	50 L/h
Desolvation gas	1000 L/h
Nebuliser gas	6 bar
Scan range	100-1200 m/z

Table S3 Scoring criteria for the arthritis scores.

Score	Symptom
0	Normal
1	Swelling of one joint (toe/wrist/ankle)
2	Swelling of more than one joint
3	Swelling of all joints
4	Skin breakdown, dysfunction or joint deformity

Table S4. Chromatographic conditions for serum metabolomic analysis.

Time (min)	Flow rate (mL/min)	Mobile phase A (%)	Mobile phase B (%)	Injection volume (μL)
0	0.4	98	2	5
2	0.4	98	2	
13	0.4	0	100	
18	0.4	0	100	
20	0.4	98	2	

Analytical conditions: Waters Acquity UPLC BEH C18 (1.7 μm, 2.1 mm × 100 mm) at 40℃ column temperature. Mobile phases: A was ultrapure water and B was acetonitrile, both of which contained 0.1% formic acid in ESI+ mode and 0.01% formic acid in ESI- mode.

Table S5. Primer sequences for the real-time PCR analysis of mRNA expression.

Gene	Forward Primer (5′-3′)	Reverse Primer (5′-3′)
Gapdh	CATCACTGCCACCCAGAAGACTG	ATGCCAGTGAGCTTCCCGTTCAG
Alox5	TACCTCAGCCTCATTGGCTCT	TGGAGCCAGTATTTGCGCTTC
Ptgs2	TTGCATTCTTTGCCCAGCACT	ACCTCTCCACCAATGACCTGAT
Cbr1	CTGGACGTGCTGGTCAACAAC	CACATTCACCACTCTGCCTTGG
ZO-1	TTGAAAGTCCACCTCCTTACAGA	CCGGATAAAAAGAGTACGCTGG
Occludin	TGTTTATGCGGACGGTGGC	GCTGTTTCCTCCATTGCTGTG
GPR41	GTGACCATGGGGACAAGCTTC	CCCTGGCTGTAGGTTGCATT
GPR109a	GGCGTGGTGCAGTGAGCAGT	GGCCCACGGACAGGCTAGGT

Table S6. Chemical composition of LPTE based on UHPLC-Q-TOF MS in positive ionization mode.

No.	Compound	Formula	Rt/min	Adducts	ppm	Fragment ions	Classification
1	Biochanin A	C16H12O5	2.70	M+Na	+7.73	322.0337, 305.0234, 295.0389, 153.0180	Flavonoids
2	5-Hydroxyflavone	C21H20O8	5.81	M+Na	+6.51	423.1082, 381.0945, 355.0819, 325.0698	Flavonoids
3	Tamarixetin	C16H14O7	4.62	M+Na	+6.89	341.0659, 287.0552, 191.0339, 243.0650	Flavonoids
4	Apigenin	C15H10O5	3.71	M+H	+1.54	271.0587, 259.0587, 229.0489, 147.0439	Flavonoids
5	Tangeritin	C20H20O7	9.13	M+H	+0.13	395.1113, 373.1286, 358.1064	Flavonoids
6	Linolenic acid	C18H30O2	9.89	M+H	+0.95	279.2318, 277.2161, 261.2212, 243.2107	Fatty Acids
7	1-Isothiocyanato-7-(methylthio)heptane	C9H17NS2	0.71	M+H	-4.73	204.0867, 202.0726, 189.0630	Others
8	Casimiroin	C12H11NO4	3.81	M+Na	+9.74	205.0496, 177.0534, 175.0384, 163.0390	Others
9	cis-Resveratrol 3-sulfate	C14H12O6S	2.49	M+K	-2.73	346.9977, 328.9878, 273.0235, 247.0178	Others
10	3,7-Dimethylquercetin	C17H14O7	2.68	M+K	+8.83	369.0399, 351.0289, 333.0184, 323.0334	Flavonoids
11	Podophyllotoxin	C22H22O8	8.55	M+H	+3.42	415.1378, 349.1101	Lignans

12	Harmalol	C12H12N2O	3.86	M+Na	-9.17	195.0644, 194.0451, 165.0553, 163.0386	Alkaloids
13	Coumesterol	C15H8O5	3.87	M+H	+6.06	251.0355, 223.0408, 269.0468, 207.0455	Flavonoids
14	Aesculetin	C9H6O4	4.12	M+H	+0.14	179.0326, 165.0181, 163.0392, 151.0391	Coumarins
15	3,3',7-Trihydroxy-4'-methoxyflavone	C16H12O6	6.21	M+H	-3.90	299.0541, 287.0548, 283.0592, 271.0605	Flavonoids
16	4-Hydroxybenzoic acid	C7H6O3	4.88	M+H	-1.54	139.0389, 123.0439	Others
17	Berberine	C20H18NO4+	5.28	M+K	+2.91	375.0844, 363.0870, 361.0710, 345.0749	Alkaloids
18	Umbelliferone	C9H6O3	4.32	M+H	-0.33	163.0389, 151.0390, 147.0436, 135.0443	Coumarins
19	Caffeine	C8H10N4O2	4.67	M+H	+0.73	195.0878, 193.0712, 138.0663, 110.0711	Alkaloids
20	Harmalan	C12H12N2	4.56	M+Na	-8.47	207.0883, 165.0544, 151.0390, 139.0387	Alkaloids
21	Rhein	C15H8O6	5.44	M+H	+2.01	285.0392, 257.0448, 239.0352, 165.0182	Others
22	Cinnamic acid	C9H8O2	5.38	M+H	-0.90	149.0596, 147.0436, 131.0497, 103.0543	Cinnamic acids
23	Formononetin	C16H12O4	5.75	M+H	-0.12	269.0804, 257.0820, 253.0848, 241.0860	Flavonoids
24	Herniarin	C10H8O3	5.20	M+H	+0.22	177.0548, 175.0393, 161.0597, 149.0594	Coumarins

25	Phloretin	C15H14O5	5.34	M+Na	+8.33	297.0753, 281.0815, 255.0654, 239.0719	Others
26	Angelicin	C11H6O3	5.35	M+H	+2.75	187.0385, 175.0388, 159.0444, 147.0439	Coumarins
27	Daidzein	C15H10O4	5.54	M+H	+3.66	255.0631, 243.0659, 241.0497, 239.0700	Flavonoids

Table S7. Chemical composition of LPTE based on UHPLC-Q-TOF MS in negative ionization mode.

No.	Compound	Formula	Rt/min	Adducts	ppm	Fragment ions	Classification
1	Hesperetin	C16H14O6	4.65	M-H	+1.52	289.0723, 287.0565, 269.0458, 205.0512	Flavonoids
2	5-Hydroxy-3,3',4',7,8-pentamethoxyflavone	C20H20O8	4.85	M-H	-1.23	369.0989, 355.0821, 343.0808, 327.0878	Flavonoids
3	Coumarin	C9H6O2	2.03	M+FA-H	-7.74	191.0336, 135.0445	Coumarins
4	Eriodictyol	C15H12O6	3.50	M+FA-H	+0.29	333.0637, 320.0558, 289.0708, 271.0601	Flavonoids
5	Gallic acid	C7H6O5	5.49	M-H	-0.20	169.0148, 125.0240	Others
6	Naringenin	C15H12O5	5.14	M-H	-0.53	271.0612, 245.0821, 229.0506, 177.0196	Flavonoids
7	Limocitrin	C17H14O8	4.32	M-H	-7.03	345.0636, 331.0465, 317.0671, 289.0723	Flavonoids
8	2-Methoxyxanthone	C14H10O3	4.88	M+FA-H	+1.50	271.0610, 211.0406, 225.0551, 227.0722	Others
9	3,3',4'5-Tetrahydroxystilbene	C14H12O4	4.64	M+FA-H	+2.42	289.0725, 245.0826, 243.0677, 225.0560	Others
10	13S-hydroxyoctadecadienoic acid	C18H32O3	8.29	M-H	-3.45	295.2268, 281.2475, 277.2175	Fatty acids
11	Quercetin	C15H10O7	6.18	M-H	+1.74	301.0359, 273.0380, 257.0459, 178.9996	Flavonoids

12	Ellagic acid	C14H6O8	4.92	M-H	+2.69	300.9998, 299.9913, 283.9967, 273.0013	Polyphenols
13	Luteolin	C15H10O6	6.17	M-H	+0.13	285.0405, 257.0455, 151.0036	Flavonoids
14	Kaempferol	C15H10O7	6.57	M-H	+0.13	285.0405, 271.0610, 269.0459, 257.0445	Flavonoids

Table S8. Identification of potential active compounds of LPTE by chemical standards.

Compound	Formula	Source	Rt/min	Adducts	ppm	Fragment ions
Quercetin	C15H10O7	LPTE	6.18	M-H	+1.74	301.0359, 273.0380, 257.0459, 178.9996
		Standard	6.14	M-H	+5.06	301.0369, 273.0410, 257.0461, 178.9996, 151.0048
Luteolin	C15H10O6	LPTE	6.17	M-H	+0.13	285.0405, 257.0455, 151.0036
		Standard	6.11	M-H	+3.64	285.0415, 257.0454, 243.0298, 151.0044
Ellagic acid	C14H6O8	LPTE	4.92	M-H	+2.69	300.9998, 299.9913, 283.9967, 273.0013
		Standard	4.92	M-H	+1.69	300.9995, 299.9911, 283.9967, 273.0054, 257.0086
Kaempferol	C15H10O7	LPTE	6.57	M-H	+0.13	285.0405, 271.0610, 269.0459, 257.0445
		Standard	6.70	M-H	+6.45	285.0423, 257.0463, 255.0309, 240.0425, 239.0362

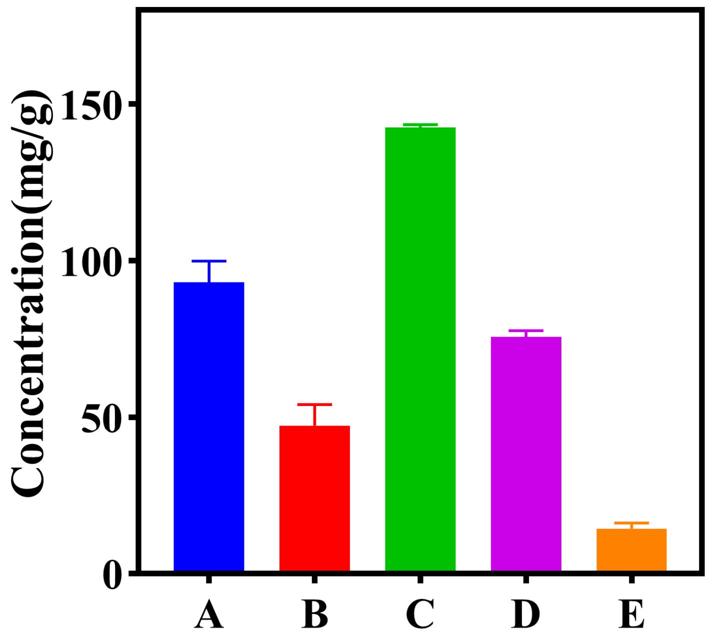

Figure S1. Determination of nutrient composition of LPTE. (**A**) Total flavonoids. (**B**) Total soluble sugars. (**C**) Total polyphenols. (**D**) Total polysaccharides. (**E**) Total free amino acids.

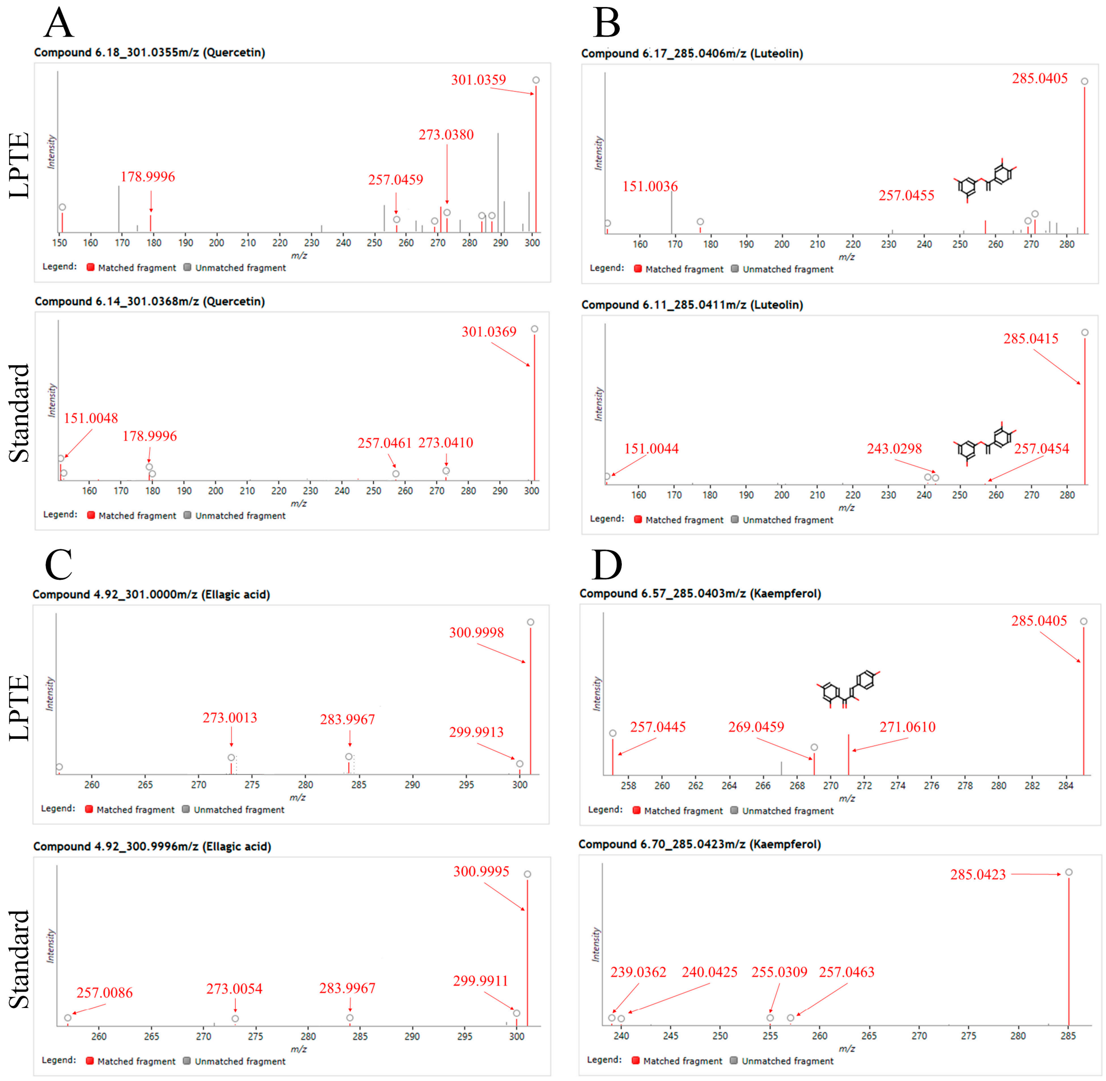

Figure S2. Mass spectrometry for the identification of potential active compounds of LPTE by chemical standards. (**A-D**) Quercetin, luteolin, ellagic acid, and kaempferol, respectively.

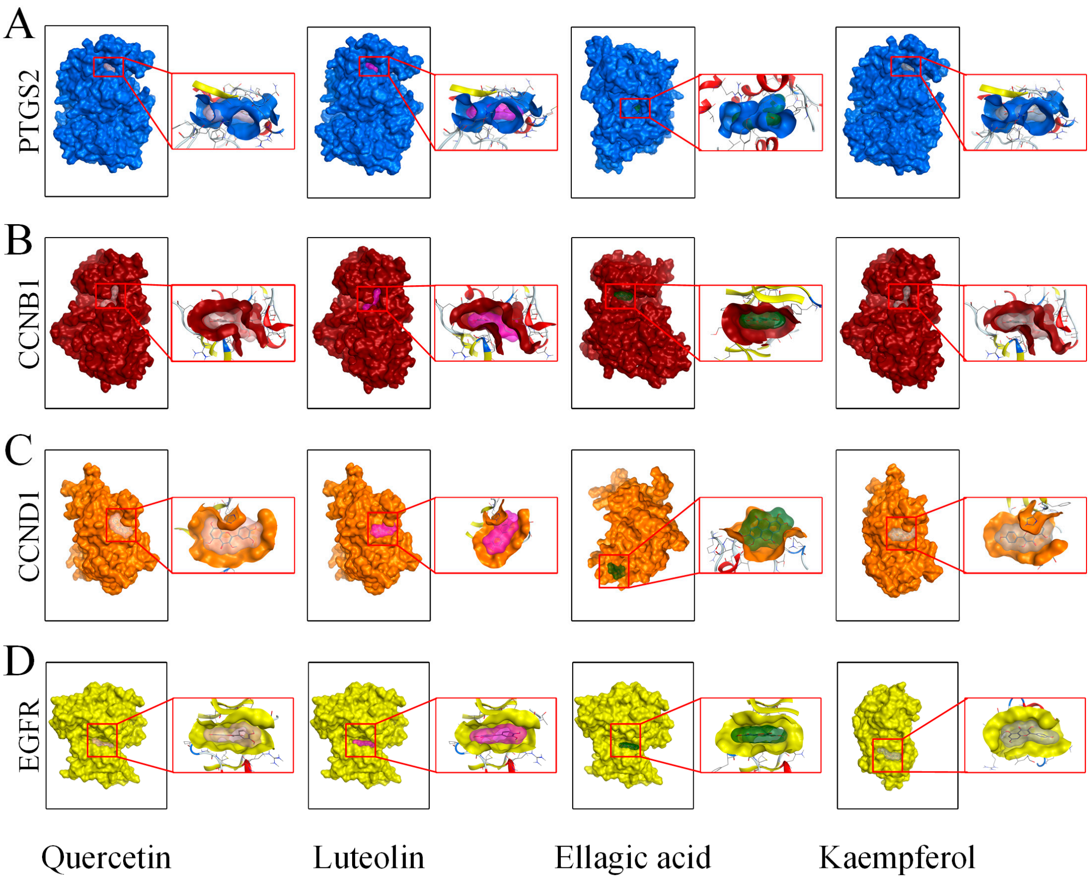

Figure S3. Potential active ingredients of LPTE docked with the core targets of RA from network pharmacology. (**A-D**) Docking model diagram of interaction between quercetin, luteolin, ellagic acid, kaempferol and PTGS2, CCNB1, CCND1, EGFR, respectively. RA, rheumatoid arthritis.

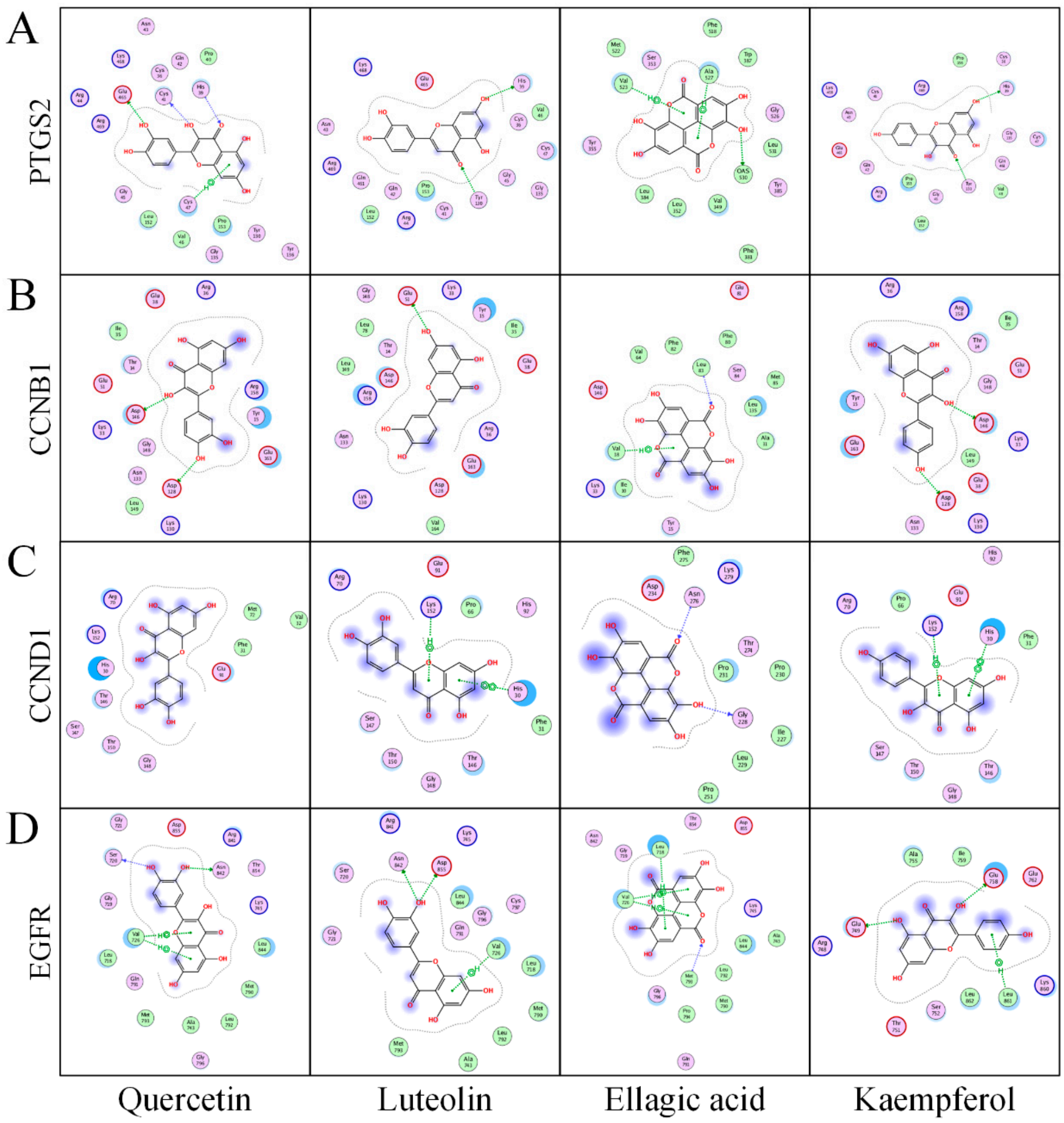

Figure S4. Interactions of selected compounds with the binding site of the hub protein. (**A**) The molecular docking poses of PTGS2-Quercetin, PTGS2-Luteolin, PTGS2-Ellagic acid, PTGS2-Kaempferol, respectively. (**B**) The molecular docking poses of CCNB1-Quercetin, CCNB1-Luteolin, CCNB1-Ellagic acid, CCNB1-Kaempferol respectively. (**C**) The molecular docking poses of CCND1-Quercetin, CCND1-Luteolin, CCND1-Ellagic acid, CCND1-Kaempferol respectively. (**D**) The molecular docking poses of EGFR-Quercetin, EGFR-Luteolin, EGFR-Ellagic acid, EGFR-Kaempferol respectively.

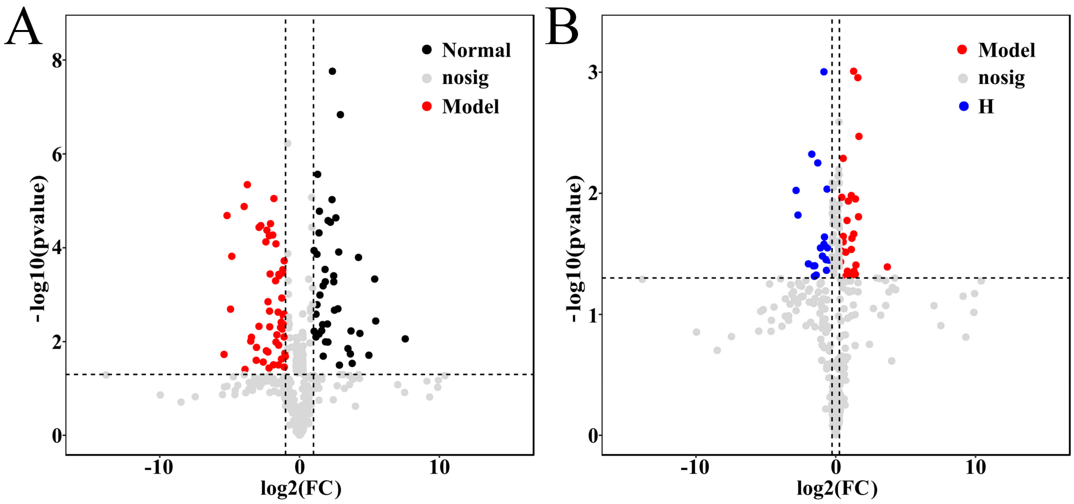

Figure S5. Volcano plot of differential metabolites. (**A**) 117 differential metabolites between the normal group and model group. (**B**) 44 differential metabolites between the model group and H group.

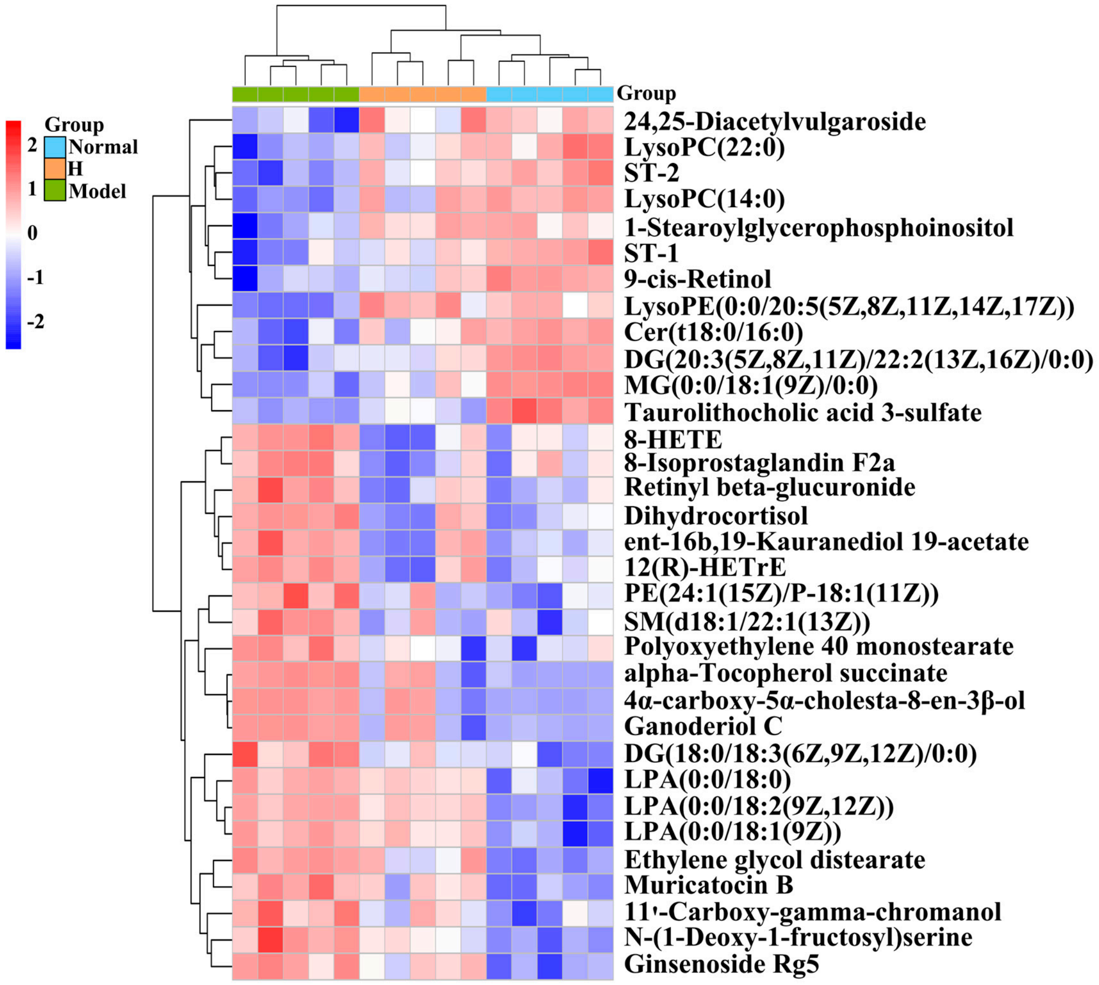

Figure S6. Heatmap of shared differential metabolites between normal *vs.* model groups as well as model *vs.* H groups.

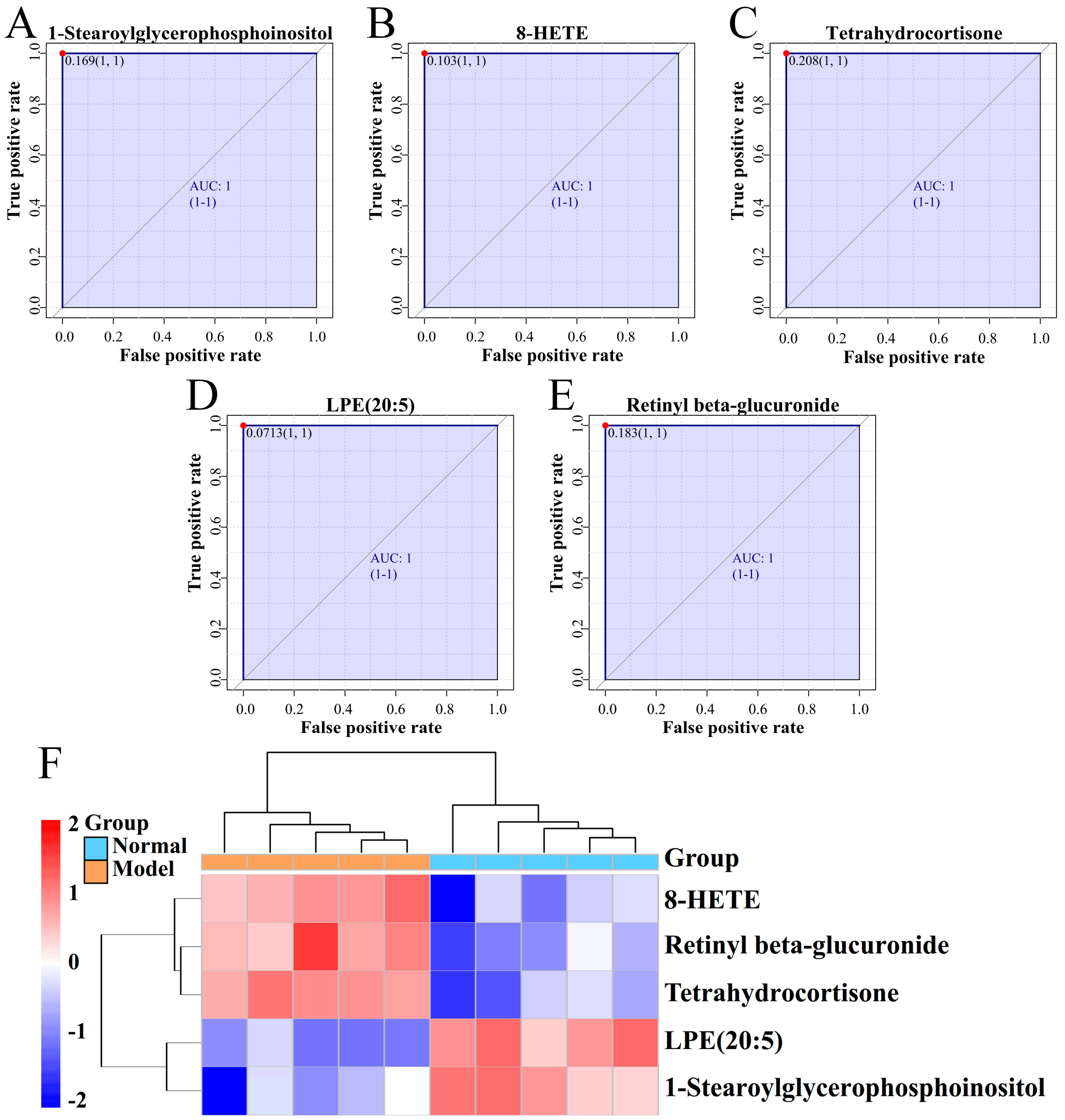

Figure S7. Assessment of diagnostic performance of 5 differential metabolites between the normal and model groups in both ESI modes.

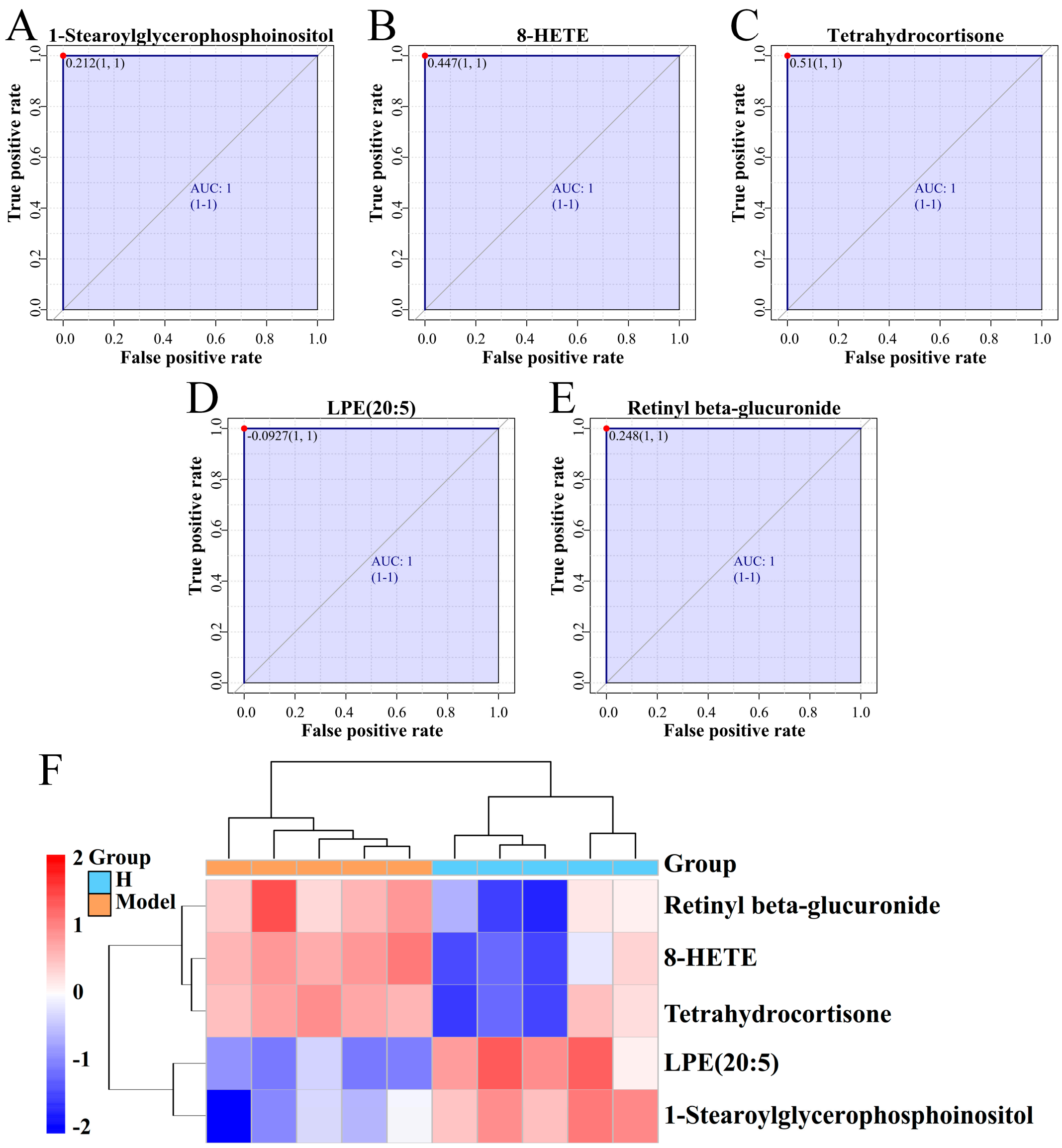

Figure S8. Assessment of therapeutic performance of 5 differential metabolites between the model and H groups in both ESI modes.

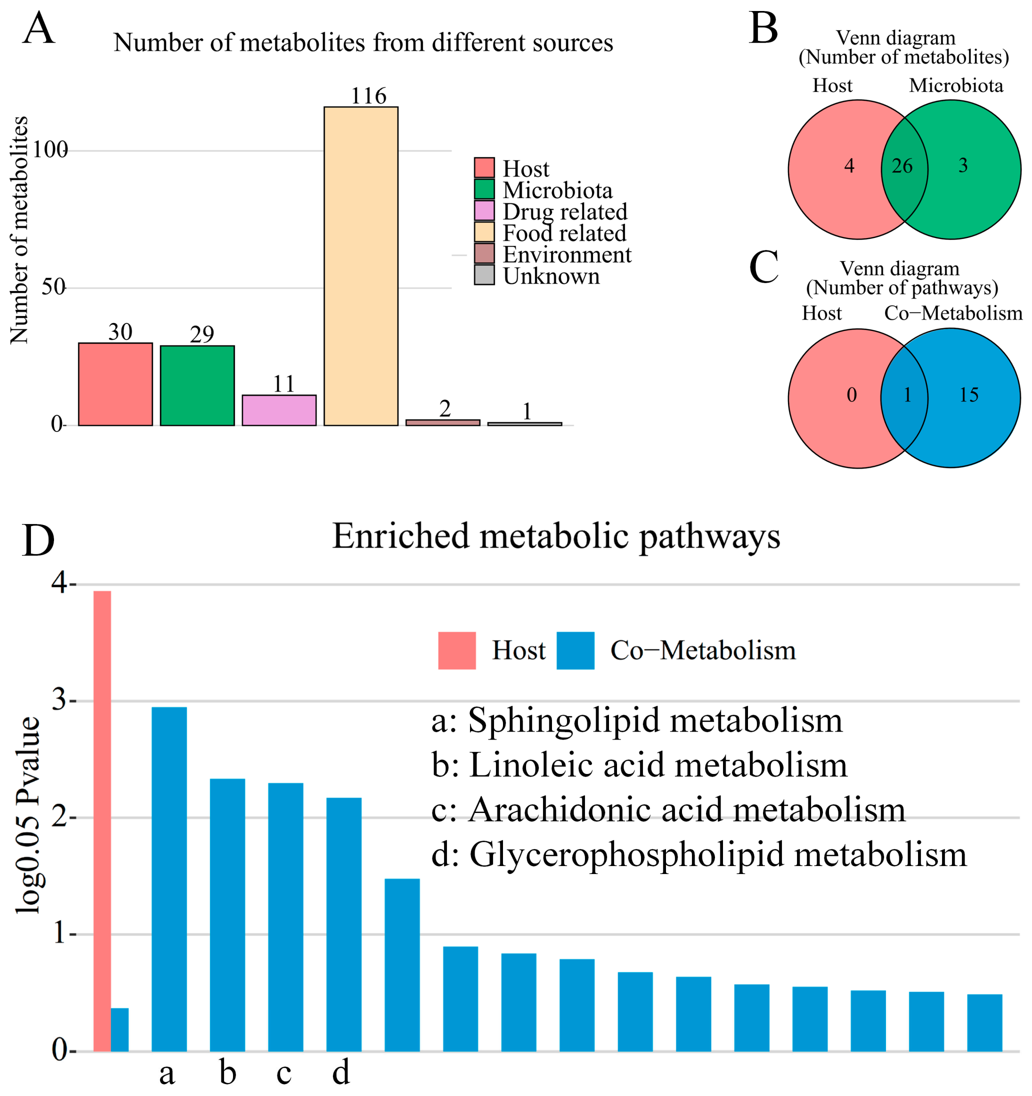

Figure S9. Metabolite traceability and metabolic function analyses by the differential metabolites between normal *vs.* model groups.

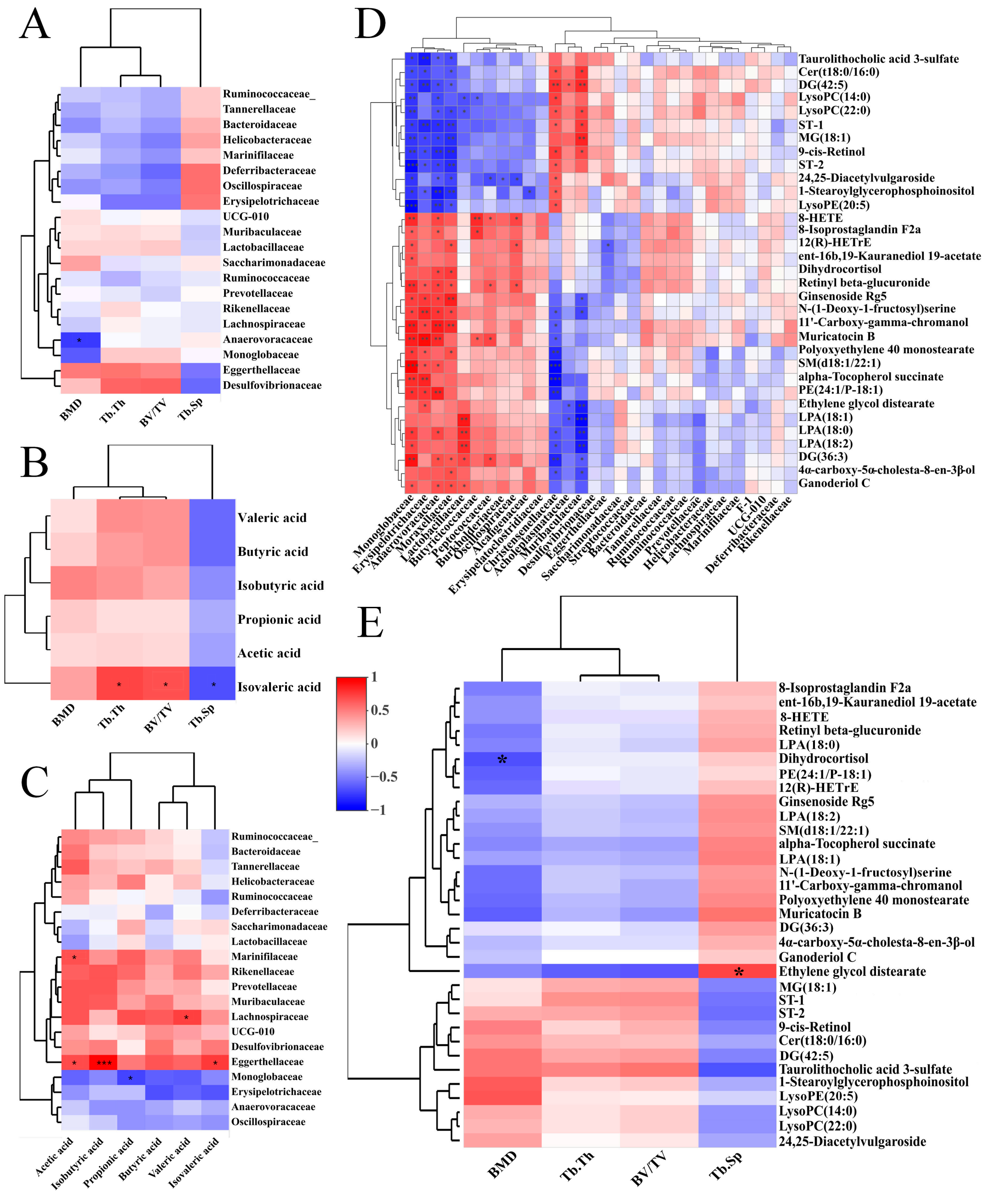

Figure S10. Heatmap of the correlation analysis among gut microbiota, serum metabolites, SCFAs, and RA-related clinical factors. (**A**) Correlation between gut microbiota and RA-related clinical factors at the family level. (**B**) Correlation analysis between SCFAs and RA-related clinical factors. (**C**) Correlation analysis between SCFAs and gut microbiota at family taxonomic level. (**D**) Correlation between metabolites and gut microbiota at the family level. (**E**) Correlation between serum metabolites and RA-related clinical factors. *, indicating significant difference ($p < 0.05$).
